# Local control and possibility of tailored salvage after hypofractionated stereotactic radiotherapy of the cavity after brain metastases resection

**DOI:** 10.1002/cam4.1486

**Published:** 2018-05-09

**Authors:** Angelika Bilger, Eva Bretzinger, Jamina Fennell, Carsten Nieder, Hannah Lorenz, Oliver Oehlke, Anca‐Ligia Grosu, Hanno M. Specht, Stephanie E. Combs

**Affiliations:** ^1^ Department of Radiation Oncology Medical Center, Medical Faculty University of Freiburg Freiburg im Breisgau Germany; ^2^ Department of Oncology and Palliative Medicine Nordland Hospital Bodø Norway; ^3^ Institute of Clinical Medicine, Faculty of Health Sciences University of Tromsø Tromsø Norway; ^4^ Deutsches Konsortium für Translationale Krebsforschung (DKTK), Partner Site Freiburg Freiburg im Breisgau Germany; ^5^ Department of Radiation Oncology, Klinikumrechts der Isar Technical University of Munich Munich Germany; ^6^ Institute of Innovative Radiotherapy (iRT) Helmholtz Zentrum Munich Oberschleißheim Germany; ^7^ Deutsches Konsortium für Translationale Krebsforschung (DKTK), Partner Site Munich Munich Germany

**Keywords:** Brain metastases, recurrence, resection cavity, salvage therapy, stereotactic radiotherapy

## Abstract

In patients undergoing surgical resection of brain metastases, the risk of local recurrence remains high. Adjuvant whole brain radiation therapy (WBRT) can reduce the risk of local relapse but fails to improve overall survival. At two tertiary care centers in Germany, a retrospective study was performed to evaluate the role of hypofractionated stereotactic radiotherapy (HFSRT) in patients with brain metastases after surgical resection. In particular, need for salvage treatment, for example, WBRT, surgery, or stereotactic radiosurgery (SRS), was evaluated. Both intracranial local (LF) and locoregional (LRF) failures were analyzed. A total of 181 patients were treated with HFSRT of the surgical cavity. In addition to the assessment of local control and distant intracranial control, we analyzed treatment modalities for tumor recurrence including surgical strategies and reirradiation. Imaging follow‐up for the evaluation of LF and LRF was available in 159 of 181 (88%) patients. A total of 100 of 159 (63%) patients showed intracranial progression after HFSRT. A total of 81 of 100 (81%) patients received salvage therapy. Fourteen of 81 patients underwent repeat surgery, and 78 of 81 patients received radiotherapy as a salvage treatment (53% WBRT). Patients with single or few metastases distant from the initial site or with WBRT in the past were retreated by HFSRT (14%) or SRS, 33%. Some patients developed up to four metachronous recurrences, which could be salvaged successfully. Eight (4%) patients experienced radionecrosis. No other severe side effects (CTCAE≥3) were observed. Postoperative HFSRT to the resection cavity resulted in a crude rate for local control of 80.5%. Salvage therapy for intracranial progression was commonly needed, typically at distant sites. Salvage therapy was performed with WBRT, SRS, and surgery or repeated HFSRT of the resection cavity depending on the tumor spread and underlying histology. Prospective studies are warranted to clarify whether or not the sequence of these therapies is important in terms of quality of life, risk of radiation necrosis, and likelihood of neurological cause of death.

## Introduction

Brain metastases are a significant cause of morbidity and mortality with an incidence of up to 40% during the trajectory of several cancer types 1, 2. New treatment options have led to improved overall survival (OS) in selected patient groups; however, the median OS of 3–6 months remains disappointing 3.

Surgery alone is known to be an effective treatment option for patients with a solitary brain metastasis, especially in those with neurological deficits. Microsurgical removal of metastases generally leads to complete remission of neurological symptoms 4. In patients undergoing surgical resection of brain metastases, the risk of local recurrence without adjuvant therapy approaches 50–60% after 1 year 5. Whole brain radiation therapy (WBRT) can significantly reduce the risk of local as well as locoregional relapse but fails to improve OS 5, 6, 7, 8.

WBRT following surgery of a limited number (1–3) of brain metastases was found to have a negative impact on quality of life 9, especially in terms of impaired neurocognitive functions 10, 11, 12. Local irradiation of the tumor bed might be a promising treatment alternative leading to significantly less neurocognitive impairment 13 while still reducing the risk of local recurrence 14, 15. Even in patients with multiple brain metastases, the use of WBRT has become more controversial and restrictive over the last years 16, 17.

In Table 1, a literature overview of postoperative stereotactic radiosurgery or stereotactic fractionated radiotherapy is given.

**Table 1:** Literature overview

Author	Year	Patients (*n*)	Dose (Gy)	Number of Fractions	1‐year Local Control (%)	Crude Local Rate (%)	1‐year OS (%)	Median OS (months)
SFRT								
Ahmed 24	2014	65	20–30	5	87	89	65	12
Bilger 14	2016	60	30–35	6–7	81.5	88.5	64.5	15
Do 31	2009	30	22–27.5	4–6	82	87	51	13 (estimated)
Minniti 32	2013	101	27	3	93	91	69	17
Specht 15	2016	46	35	7	88	87	76	25
Steinmann 33	2012	33	30–40	5–10	73	76	64	20
Ling 34	2015	99	10–28	1–5	72	Not available	55	12
Wang 35	2012	35	24	3	80	Not available	Not available	6
SRS								
Brennan 36	2014	49	18	1	78	70	Not available	Not available
Do 31	2009	30	15–18	1	82	87	51	13 (estimated)
Hartford 37	2013	47	12–20	1	86	66	52	13 (estimated)
Hwang 38	2010	25	15–20	1	Not available	100	Not available	15
Iwai 39	2008	21	13–20	1	82	76	Not available	20
Jagannathan 40	2009	47	19	1	Not available	76	Not available	12
Jensen 41	2011	106	17	1	80	87	47	11
Karlovits 42	2009	52	15	1	82	82	Not available	15
Kelly 43	2010	17	15–18	1	Not available	89	93	12
Limbrick 44	2009	15	16–24	1	Not available	73	Not available	20
Luther 45	2013	120	11–20	1	86	87	Not available	Not available
Mathieu 46	2008	40	16	1	Not available	73	Not available	13
Ogiwara 30	2012	56	14–20	1	Not available	91	75 (estimated)	20
Prabhu 47	2012	62	15–21	1–4	78	83	53	13
Soltys 48	2008	65	18	1	79	86	57	15
Mahajan 20	2017	64	12–16	1	72	76	ca. 70	17
Brown 19	2017	98	20–24	1	60.5	Not available	ca. 50	12

A retrospective multicenter study (Department of Radiation Oncology, University Medical Center Freiburg and Department of Radiation Oncology, University Hospital Technical University Munich) was recently performed to evaluate the role of hypofractionated stereotactic radiotherapy (HFSRT) in patients after surgical resection of ≤3 brain metastases. Local control (LC) after 12 months was 75%. The main advantages of HFSRT of the resection cavity in comparison with WBRT are the short treatment time, the high probability of local control, and the reduced risk of normal tissue reactions including neurocognitive impairment 13, 14, 15. Detailed outcome data have already been presented elsewhere 18.

Importantly and in contrast to adjuvant WBRT, adjuvant HFSRT does not influence distant intracranial progression. Moreover, several dose prescription and fractionation concepts exist with different target volume guidelines 14, 15, 19, 20.

For example, some centers use single‐dose SRS as adjuvant treatment and the dose prescription is depending on the size of the resection cavity 19, 20. Others use HFSRT as adjuvant treatment, and the total dose is in some institutions depending on the absence or presence of residual tumor 14, 15.

It is therefore necessary to evaluate the feasibility of salvage therapy and the risk of side effects, especially in cases of reirradiation. In the present retrospective multi‐institutional study, the patterns of intracranial local (LF) and locoregional (LRF) failure and the salvage treatment strategies for progression were evaluated.

## Materials and Methods

### Patient characteristics

We investigated 181 patients with newly diagnosed brain metastases who were treated with HFSRT following resection (July 2009 to November 2015) (see Table 1). Surgical resection was performed to palliate neurological dysfunctions (e.g., seizures or motor deficits), intracranial pressure, or if the diameter of the lesion exceeded 3 cm. Patients prognosis was assessed according to the graded prognostic assessment (GPA) score and the recursive partitioning analysis (RPA) classes 21, 22.

Median age was 62 years (range 18–85 years), 99 (55%) patients were men, and 82 (45%) patients were women. The most common primary tumors were non‐small‐cell lung cancer (NSCLC) (36%), gastrointestinal cancer (16%), and breast cancer (15%). The patient characteristics are summarized in Table 2.

**Table 2:** Patients’ characteristics

	*n*	%
Age		
62 (19–85)	181	100
Sex		
Female	82	45.3
Male	99	54.7
Primary tumor		
NSCLC	66	36.5
Gastrointestinal cancer	28	15.5
Breast	29	16
Malignant melanoma	20	11
RCC	5	2.8
Sarcoma	2	1.1
Others	31	17.1
Radioresistant tumors	27	14.9
Mean time from first diagnosis of primary tumor to first diagnosis of brain metastases (months)	33.5 (0–288)	
Synchronous BM (0–1 months)	63	35
Metachronous BM (>1 month)	118	65
Resection status (MRI <48 h postop)	74	
Complete resection	49	66.2
Residual tumor	25	33.8
Resection status (Planning MRI)	180	
Complete resection	135	75
Residual tumor	45	25
Number of lesions		
1	155	85.6
≥2	26	14.4
RPA class		
1	37	20.4
2	132	72.9
3	12	6.6
GPA score		
1	8	4.4
1.5–2.5	111	61.3
3	31	17.1
3.5–4.0	31	17.1
Mean resection cavity size (cm^3^)		
16 (0.9–114.2)		
Mean planning target volume size (cm^3^)		
38.8 (3.5–205.1)		

**Figure 1:**
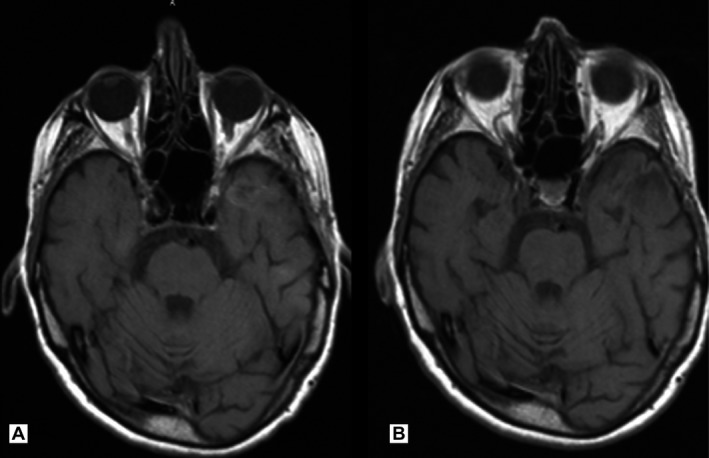
(A) Patient after resection of a renal cell carcinoma brain metastasis of the left temporal lobe. (B) The same patient 4 years after HFSRT of the resection cavity with complete remission.

In our initial analysis 18, we evaluated LC, distant intracranial control, OS, and progression‐free survival. Figure 1 shows two CMRIs of a patient treated with HFSRT before treatment and 4 years after treatment without local progression.

The present analysis focused on individual treatment strategies after intracranial recurrence. We evaluated different salvage therapies after local or locoregional recurrences.

The present work complies with the principles laid down in the Declaration of Helsinki and was approved by the appropriate ethical committees in both participating institutions.

### Radiation therapy

Radiotherapy planning protocols of HFSRT to the resection cavity according to the different institutional standards were published before 14, 15.

#### Treatment planning—Munich

Individual fixation using a thermoplastic mask system for stereotactic setup was used for each patient with the Brainlab**©** mask system. For all patients, target volume definition was based on CT and MRI; generally, the postsurgical MRI was used for treatment planning; a dedicated planning MRI was acquired in patients where the time interval between surgery and postoperative HFSRT exceeded 2 weeks 15. The gross tumor volume (GTV) was defined as any residual tumor; the clinical target volume (CTV) consisted of the GTV and the resection cavity plus a safety margin of 2–3 mm accounting for potential microscopic spread. The PTV (planning target volume) was defined as the CTV expanded with a 1 mm margin. A median dose of 35 Gy in seven fractions (BED 52.5 Gy, alpha/beta value 10 Gy), 5 Gy each was applied, with daily image‐guided radiotherapy (IGRT) by robotic ExacTrac positioning (Brainlab, Germany) on a linear accelerator (LINAC) with a Micro‐MLC (Varian, Palo Alto, CA) and 6 MeV photons.

#### Treatment planning—Freiburg

Patients were immobilized using a thermoplastic mask system. HFSRT target volume definition was based on CT and MRI 14. In patients with residual/recurrent tumor, the GTV was delineated on contrast‐enhanced MRI. Residual/recurrent tumor was defined as contrast enhancement adjacent to the resection cavity. The CTV was defined as the contrast enhancement/GTV and resection cavity plus 1 mm. The PTV was defined as CTV expanded with a 2 mm margin. The treatment was delivered either by dynamic conformal arcs or by intensity‐modulated radiotherapy using a Varian TrueBeam STX Brainlab Novalis LINAC with 6 MeV photons. The median prescribed dose was 30 Gy in six fractions (BED 45 Gy, alpha/beta value 10 Gy) in patients with complete tumor resection and 35 Gy in seven fractions (52.5 Gy, alpha/beta value 10 Gy) in patients with residual/recurrent tumor after surgery.

### Follow‐up

All patients were followed up regularly including contrast‐enhanced brain imaging as well as clinical follow‐up, initially 6 weeks after treatment, then in 3‐month intervals. After two years of recurrence‐free follow‐up, the intervals were prolonged individually. In case of local or distant intracranial failure, salvage therapy was performed after interdisciplinary discussion (neurosurgical intervention with/without adjuvant radiation therapy performed as WBRT or radiosurgery).

### Endpoints

LF was defined as new contrast enhancement in the previously irradiated tumor bed or increasing residual/recurrent tumor volume in MRI. Radiation‐induced changes (including radiation necrosis) were excluded based on serial follow‐up and related clinical course. Lesions with increased contrast enhancement and edema after 6 weeks of treatment with corticosteroids and pentoxiflline for potential radiation‐induced damage were classified as tumor progression; reduced edema and contrast enhancement were diagnosed as treatment‐related changes/radiation necrosis. In case of severe neurological symptoms, an operation was performed to obtain a histological diagnosis.

Toxicities were classified as acute, if they occurred during treatment or up to the first 6 weeks after the end of radiation. If they occurred later, toxicities were considered to be late toxicities. As neurocognitive impairment was not measured using standardized tests, it has not been included in this evaluation. Symptomatic brain necrosis was assessed by follow‐up MRI and reviewed by a multidisciplinary team including the treating radiation oncologist, neurosurgeon, neuroradiologist, and pathologist. If there was a progressive, contrast‐enhancing lesion and symptoms led to an impaired quality of life, surgical resection was performed after a consensus statement was obtained in an interdisciplinary tumor board. If histopathological examination of the resection specimen revealed residual tumor, the case was considered as local recurrence. If not, it was considered as post‐therapeutic brain necrosis. LRF was defined as new brain metastases or leptomeningeal enhancement outside the previously irradiated volume.

### Statistics

The median follow‐up time for analysis of OS was 12.6 months after HFSRT of the resection cavity (range 0.3–80.2 months). In 159 patients, follow‐up imaging for the analysis of LC and PFS was available. The median follow‐up time between the start of HFSRT and the last available cranial imaging was 10.7 months (range 1.1–70.1 months). Data from both institutions were pooled in a dedicated database. All statistical calculations such as Kaplan–Meier analyses were performed using SPSS Statistics version 23 (IBM, New York, NY). A *P*‐value ≤ 0.05 was considered statistically significant.

## Results

Crude local control after HFSRT of the resection cavity was 80.5%. Actuarial Kaplan–Meier analysis revealed a one‐year local control rate of 75% and a two‐year local control rate of 70% (Fig. 1 presents a patient`s resection cavity with 4‐year event‐free survival).

Female patients had a numerically higher local control rate than male patients (81% vs. 68%, *P* = 0.237).

Resection cavities >11.7 cm^3^ were at significantly increased risk of local recurrence (one‐year local recurrence rate 68% vs. 82% for small cavities, *P* = 0.033). Detailed outcome data have already been presented elsewhere 18.

### Initial salvage treatment after HFSRT

A total of 100 of 159 (63%) evaluable patients experienced intracerebral tumor recurrence. Median time to the first intracranial recurrence was 5 months (6 LF, 73 LRF, 21 LF+LRF).

At the time of first recurrence, 81 of 100 patients (81%) received salvage therapy. A total of 14 patients underwent repeat brain surgery, and 78 patients received repeat radiotherapy. Of those, 41 patients with multiple (more than four) new brain metastases (53%) received WBRT. Patients with single or maximum four metastases distant from the site of initial HFSRT or a history of previous WBRT were retreated by HFSRT (14%) or stereotactic radiosurgery (SRS, 33%). Table 3 shows an overview of the salvage treatments.

**Table 3:** Local failure and overview of salvage therapy in cases of recurrence after resection and postoperative HFSRT of brain metastases

	First Recurrence	Second Recurrence	Third Recurrence	Fourth Recurrence
Intracranial recurrence (*n*)	100	48	12	5
Localization of tumor Recurrence (*n*)				
LF	6	5	2	
LRF	73	38	8	3
LF + LRF	21	5	2	2
Retreatment (*n*)	81	32	6	3
OP (+/−RTx)	14 (11/3)			
RTx	78			
WBRT	41	10	1	2
SRS	26	18	4	1
HFSRT	11	4	1	
Time interval to initial HFSRT (months)	5 (1–27)	10 (2–42)	14 (11–55)	23 (14–28)

LF, local failure; LRF, locoregional failure; WBRT, whole brain radiotherapy; SRS, stereotactic radiosurgery; HFSRT, hypofractionated stereotactic radiotherapy.

### Further salvage treatment

Forty‐eight patients experienced a second intracranial failure. A total of 32 of 48 patients (67%) received further salvage therapy (10 WBRT, 18 SRS, and four HFSRT). Median time to second recurrence was 10 months (five LF, 38 LRF, and five LF+LRF).

Twelve patients developed a third failure (two LF, eight LRF, and two LF+LRF) after a median time of 14 months, and six of 12 (50%) had further radiotherapy (one WBRT, four SRS, and one HFSRT).

After a median time of 23 months, five patients had a fourth recurrence (three LRF and two LF+ LRF) and three of five (60%) had another salvage treatment (two WBRT and one SRS). Overall, three patients received five consecutive courses of radiotherapy.

A total of 54 of 159 patients (34%) eventually received WBRT during follow‐up, 32 of 159 received another HSFRT (20%), and 33 of 159 (21%) received SRS as salvage treatment.

### Salvage treatment of local recurrence

During follow‐up, a total of 31 patients (19.5%) developed a local recurrence within the initial treatment field. Twenty‐seven of 31 patients (87%) showed a local recurrence or a combined local and distant intracranial progression at first follow‐up. Of those, 20 of 27 (74%) patients were effectively treated with salvage therapy. At the time of second intracranial recurrence, three new local recurrences occurred (10 in total, seven persisted after ineffective or omitted salvage therapy). A total of seven of 10 (70%) recurrences were salvaged successfully at the time of second recurrence. At the time of third intracranial failure, one more local recurrence (three patients with persisting local failure) occurred. Two of four (50%) recurrences were salvaged successfully. At the time of fourth intracranial recurrence, two patients showed persisting local recurrence; no new local recurrence occurred.

Overall, further radiation treatment was used as most common salvage strategy. Surgery was only used in case of severe symptoms from the metastases.

Eight (4%) patients in the whole cohort experienced radionecrosis at the initial treated resection cavity (single course of radiation). After steroid/pentoxiflline treatment showed no clinical benefit, in seven of those patients, the contrast‐enhancing lesion was removed by neurosurgical intervention and the histopathological evaluation of the resected specimen showed only necrotic tissue and no sign of viable tumor cells. The mean PTV size of the seven patients with confirmed radionecrosis was 26.23 cm^3^ (range 17.5–33.75 cm^3^) and was not bigger than the mean PTV of all patients included in the study (mean 38.8 cm^3^, range 3.5–205.1 cm^3^). Three of these patients had breast cancer, one had a renal cell carcinoma, one had rectum carcinoma, one had lung cancer, and one had a thymus carcinoma as primary tumor. After operation of the radionecrosis, five of seven (71%) patients died during follow‐up. The mean time from operation of the radionecrosis to death was 14.4 months (range 7–27 months), which means that surgery was successful in preventing death from radionecrosis.

No other cases of severe treatment‐related side effects (CTCAE≥3) were observed. Especially, no bleedings or seizures occurred.

## Discussion

At two tertiary care centers in Germany, we performed a retrospective study to evaluate the role of hypofractionated stereotactic radiotherapy (HFSRT) in patients with brain metastases after surgical resection. Local control (LC) after 12 months was 75% 18. In the present analysis, we focused on salvage therapy of brain metastases in case of intracranial progression after HFSRT of the resection cavity. We were interested in finding out whether or not salvage treatment after HFSRT is possible without leading to a high risk of radiation necrosis. In particular, need for salvage treatment, for example, WBRT, surgery, or stereotactic radiosurgery (SRS), was evaluated. Both intracranial local (LF) and locoregional (LRF) failure were analyzed.

A total of 100 of 159 (63%) patients received salvage therapy (surgery with or without adjuvant radiation therapy, WBRT, and SRS). Local failures were rare, and distant intracranial failures were often effectively salvaged by further radiotherapy. Even most local failures were salvaged successfully.

Previously, we have shown that postoperative HFSRT to the resection cavity in patients with brain metastases is a highly effective concept, leading to satisfactory long‐term local control after surgery. Our data for local control were even better than in the two prospective studies published in 2017 about radiation therapy of the resection cavity 19, 20 (see Table 1, literature overview). Many reasons might explain this fact, including significant differences in target volume definition with respect to safety margins for the CTV. The most recent randomized trials offered radiosurgery, with very small to no safety margins accounting for microscopic spread 19, 20. In our multicenter setting, both groups applied at least 2–3 mm safety margins from GTV to CTV. This might have contributed to the higher rate of local control observed in our study.

New guidelines for contouring for postoperative cavity SR/HFSRT are needed. Soliman et al. have tried to find a consensus for contouring, but clinical data to support these recommendations are still missing 23. For certain histologies, the fractionated concept, albeit hypofractionated, is also more promising in terms of radiation biology compared to the single‐dose approach reported by others 24.

On the other hand, as this is a retrospective analysis, it appears possible that prospective follow‐up depicts more failures, and other sources of selection bias are possible, too.

It has been shown previously that male sex and large resection cavity size (>11.7 cm^3^) are predictors for inferior overall local control 18. Therefore, in future prospective trials, a higher dose for large cavity sizes might be discussed. However, large volume bears a higher risk for radiation‐related side effects which must be kept in might in those considerations. As also previously shown, there was a trend toward earlier intracerebral progression if the first diagnosis of brain metastases was synchronous to the first diagnosis of the primary tumor. For patients with more than one brain metastasis at the time of HSFRT, there was also a tendency toward earlier intracerebral recurrence.

The data in the present manuscript suggest that, after local multimodal therapy for brain metastases (surgery + local RT), WBRT can be deferred without compromising survival. As WBRT may reduce neurocognitive function, leading to worsening of quality of life, without improving OS 5, 6, 7, 8, WBRT can be withheld and performed in cases of multiple new metastases. Previously, Gorovets et al. published a nomogram to predict the time between SRS and WBRT salvage treatment 25. Also, in case of HFSRT, this nomogram could be used to decide which patients benefit most of HFSRT to the resection cavity instead of WBRT, if further studies confirm its validity in this setting. Even in patients with multiple brain metastases, standard WBRT could be replaced by WBRT with hippocampal sparing, depending on expected survival, in order to reduce toxicity 26.

Decision‐making requires thorough assessment of the patients’ general state of health and expected survival. Some patients with multiple brain metastases and terminal illness will not benefit from WBRT because they are in a very palliative situation 27. Newer review articles and articles about risk analysis of distant brain failures after radiosurgery could support decision‐making 16, 17. In general, a trend toward a more restrictive use of WBRT in favor of local treatment with SRS even in patients with multiple brain metastases is seen, but also socioeconomic and racial/ethnic disparities in the use and uptake of SRS in the USA 28.

As we performed a retrospective study, the decision about the modality of salvage therapy (SRS/surgery or WBRT) was made by the physician in charge. In general, patients with single or maximum four metastases distant from the site of initial HFSRT or a history of previous WBRT were retreated by HFSRT or SRS. However, it might be possible that also patients in good clinical condition with >4 metastases distant from the site of initial treatment could benefit from a localized SRS as salvage therapy.

Although, in HFSRT, the irradiated volume is bigger compared to SRS, repeated radiotherapy or surgery did not lead to a high rate of radionecrosis in our study.

Due to the high rate of distant intracranial relapse, regular follow‐up with MRI is mandatory. Whether this strategy of repeat imaging and treatment results in cost‐effectiveness and a satisfactory number of quality‐adjusted life years compared to up‐front WBRT remains to be established.

As we have shown in this analysis deferred salvage with WBRT/SRS and surgery is feasible and appears also safe and efficient, but discrimination between recurrence and treatment‐induced side effects is sometimes challenging and often requires additional imaging. Furthermore, a retrospective study cannot capture all aspects of brain toxicity. In addition, short follow‐up and early death events may decrease the apparent risk of late toxicity 29. Despite these caveats, even patients with four salvage radiation treatments had no excessive risk of radiation necrosis.

In an analysis by Ogiwara et al. 30, who looked at patterns of recurrence after SRS to the resection cavity, similar results concerning salvage treatments were found, but our work represents the largest cohort (two German institutions) to our knowledge in the literature. Prospective studies concerning HFSRT of the resection cavity are currently being carried out, which will shade more light on subsequent treatment utilization and sequelae.

Tailored salvage treatment after HFSRT to the resection cavity using SRS, WBRT, or surgery appears safe and efficient. As toxicity was recorded retrospectively and without capturing all domains, prospective studies are warranted. Currently, the LAUREL study at the Technical University of Munich (TUM) is evaluating toxicity and outcome regarding imaging response, patterns of failure, and survival in patients with brain metastases treated with HFSRT. Due to the high rate of distant intracranial failure after local multimodal therapy for brain metastases, regular follow‐up with MRI is mandatory.

## Ethical Approval

All procedures performed in studies involving human participants were in accordance with the ethical standards of the institutional and/or national research committee and with the 1964 Helsinki declaration and its later amendments or comparable ethical standards. For this type of study, formal consent is not required.

## Conflict of Interest

The authors state that they have not published or submitted the manuscript elsewhere. All authors state that they have no conflict of interest.
